# Crucial role of pro-inflammatory cytokines from respiratory tract upon PM_2.5_ exposure in causing the BMSCs differentiation in cells and animals

**DOI:** 10.18632/oncotarget.23158

**Published:** 2017-12-11

**Authors:** Xiaoting Jin, Ruijun Su, Ruijin Li, Long Cheng, Zhuoyu Li

**Affiliations:** ^1^ Institutes of Biomedical Sciences, Shanxi University, Taiyuan, China; ^2^ Institute of Biotechnology, Key Laboratory of Chemical Biology and Molecular Engineering of National Ministry of Education, Shanxi University, Taiyuan, China; ^3^ Institute of Environmental Science, Shanxi University, Taiyuan, China; ^4^ Department of Neurology, Harvard Medical School, Boston, MA, USA

**Keywords:** PM_2.5_, BMSCs, inflammation, ROS, mitochondria

## Abstract

Fine particulate matter exposure may cause health risk, including cardiovascular diseases and cancer. Bone marrow mesenchymal stem cell (BMSC), a typical model for evaluating pollutant toxicity, has been closely linked to these diseases, due to its characteristics of differentiation. We therefore studied the BMSCs differentiation and its roles in inflammatory activation in the respiratory tract upon PM2.5 exposure using both *in vitro* and *in vivo* models. BMSCs differentiation into endothelial-like cells (ELCs) and cancer-associated fibroblasts cells (CAFs) was enhanced in response to conditioned medium from PM_2.5_–treated 16HBE cells. PM2.5 elevated inflammatory cytokines’ expression and secretion in 16HBE cells. However, induction of differentiation markers was reduced when IL-1β, IL-6 and COX-2 neutralizing antibodies were added to the conditioned medium. Furthermore, PM_2.5_ induced ROS formation and NADPH oxidase (NOX) expression in 16HBE cells. DPI (inhibitor of ROS from NOX) or NAC (inhibitor of ROS) supplement reduced PM_2.5_-induced inflammatory activation and BMSCs differentiation. Likewise, a concomitant disorder of mitochondrial morphology and respiratory chain was observed. In addition, Rot or AA (inhibitor of mitochondrial complex I or III) supplement restored PM2.5-induced toxic effects. Moreover, the results coincided with the *in vitro* data obtained from SD rats post-exposed to different doses of PM_2.5_ for 30 days. PM_2.5_ enhanced the BMSCs differentiation and inflammatory cytokines’ expression in respiratory organs of SD rats, including lung and trachea tissue. This study uncovers that PM_2.5_ promotes the BMSCs differentiation via inflammatory activation mediated by ROS induction from NOX and mitochondria in the respiratory tract.

## INTRODUCTION

Dust-haze is a continuing world-wide challenge to public health, and has become a global concern [1]. Fine particulate matter (PM_2.5_; aerodynamic diameter less than 2.5 μm) is readily inhaled by the human body and deposited in the respiratory system. It is marked by remote transportation distance, longer retaining duration and no filtering resistance due to its physical construction [2]. Thus, PM_2.5_ has drawn great concern and posed a serious threat to human health due to potential bio-accumulation [3, 4]. However, since its complexity of components and way of exposure, including endogenous exposure and exogenous exposure, the relevant toxic mechanisms are poorly understood. Accordingly, the appropriate biological and/or cellular models, along with the actual environmental dose and exposure method are crucial for research of particulate matter toxicology and relevant mechanisms.

BMSCs, the ancestors of many cells, is a typical and important model for evaluating the health risk and pollutant toxicity, due to its features and characteristics of differentiation. As BMSCs regulate hematopoietic stem cell development and maturation, it has been linked to cardiovascular diseases and cancer [5]. Therefore, it is important to understand if PM_2.5_ exposure has a modulatory roles in the BMSCs differentiation. Specifically, BMSCs function as precursors for vascular endothelial-like cells (ELCs), which participate in the construction of functional vascular structures, promote angiogenesis in tumor tissues and provide nutrients for tumor growth [6]. In addition, BMSCs can differentiate into cancer-associated fibroblasts cells (CAFs), a type of tumor-associated stromal cells involved in tissue construction [7]. Current studies of effects of PM_2.5_ exposure on BMSCs differentiation are limited, and mainly focus on endothelial progenitor cells (EPCs), which play a critical role in maintaining the structural and functional integrity of vasculature. It has been reported that PM_2.5–10_ exposure significantly suppressed the number and function of stem cells (SCs) and EPCs in animals and humans [8–10]. Nevertheless, the mechanisms for the detrimental effects of PM on EPCs remain to be fully defined. Existing studies have adopted the direct exposure method, which can not reflect the actual pathway of PM_2.5_ exposure. Additionally, there are no reports about the correction between PM_2.5_ exposure and BMSCs differentiation into ELCs and CAFs. Hence, it needs further investigation and an in-depth mechanistic analyses.

When inhaled by the human body, most PM_2.5_ particles are easily deposited in the respiratory tract and alveolar area, which have rich capillary networks due to their large surface areas. Upon inhalation, PM_2.5_ particle is phagocytosed and different diffusive cytokines are secreted from the respiratory tract tissues, which spread to the blood system, altering human's microenvironment and then resulting in the damage to human health [11]. Therefore, effects of cytokines secreted from PM_2.5_-stimulated respiratory tract on human health can not be ignored. Inflammatory cytokines are an important and typical secretion caused by PM_2.5_ exposure [12, 13]. While there were burgeoning data indicated that inflammatory cytokines can promote the BMSCs differentiation, and it was a main mechanism in PM_2.5_ exposure-induced human disease, including cancers [14, 15], little is known in the case of PM_2.5_-exposed respiratory tract. Therefore, we build *in vitro* cell model to explore the effects of PM_2.5_-stimulated respiratory secretions on BMSC differentiation.

Several *in vitro* and *in vivo* studies have documented that reactive oxygen species (ROS) can regulate expression of inflammatory cytokines, and that it had vital roles in PM_2.5_-mediated adverse health on the human body [16, 17]. Mitochondria are accountable for the generation of ROS, which is generated with the leak of electron mainly from mitochondrial respiratory chain complexes I and III [18]. Additionally, NADPH oxidases (NOXs) are a cell membrane-bound proteins and the other main source of cellular ROS (Lambeth 2004). While PM_2.5_ exposure induced the ROS generation by affecting NOX expressions or mitochondria disorders [19, 20], there is no fully comparison between their contribution to the response. Consequently, we intend to analyze the reason for PM_2.5_-stimulated secretions from respiratory tract, and focus on two main causes of ROS, including NADPH oxidases and mitochondria.

Due to extensive vehicle exhaust emissions and coal combustions in residential stoves for cooking and heating, northern Chinese cities face serious problems of PM_2.5_ pollution, particularly during winter [21]. This situation is worsening with the urbanization and industrialization of Taiyuan, northern city of China and a center for coal-based electricity production and many chemicals industries [22]. This current study was designed to expose the SD rats to PM_2.5_ at the actual environmental concentration and analyze the risk of BMSCs differentiation into ELCs and CAFs. According to the main pathway of PM_2.5_ entered to the bone marrow, the *in vitro* model was established, and the roles of inflammatory cytokines secreted from the PM_2.5_-stimulated respiratory tract in the differentiation of BMSCs and its possible mechanism were addressed. Our findings provide understanding about the detrimental effect of these cytokines on stem cell differentiation, and reveal a mechanistic and theoretical basis for preventing outcomes in polluted environments and environmental toxicology.

## RESULTS

### Characterization of winter PM_2.5_ in Taiyuan

**Figure 1:**
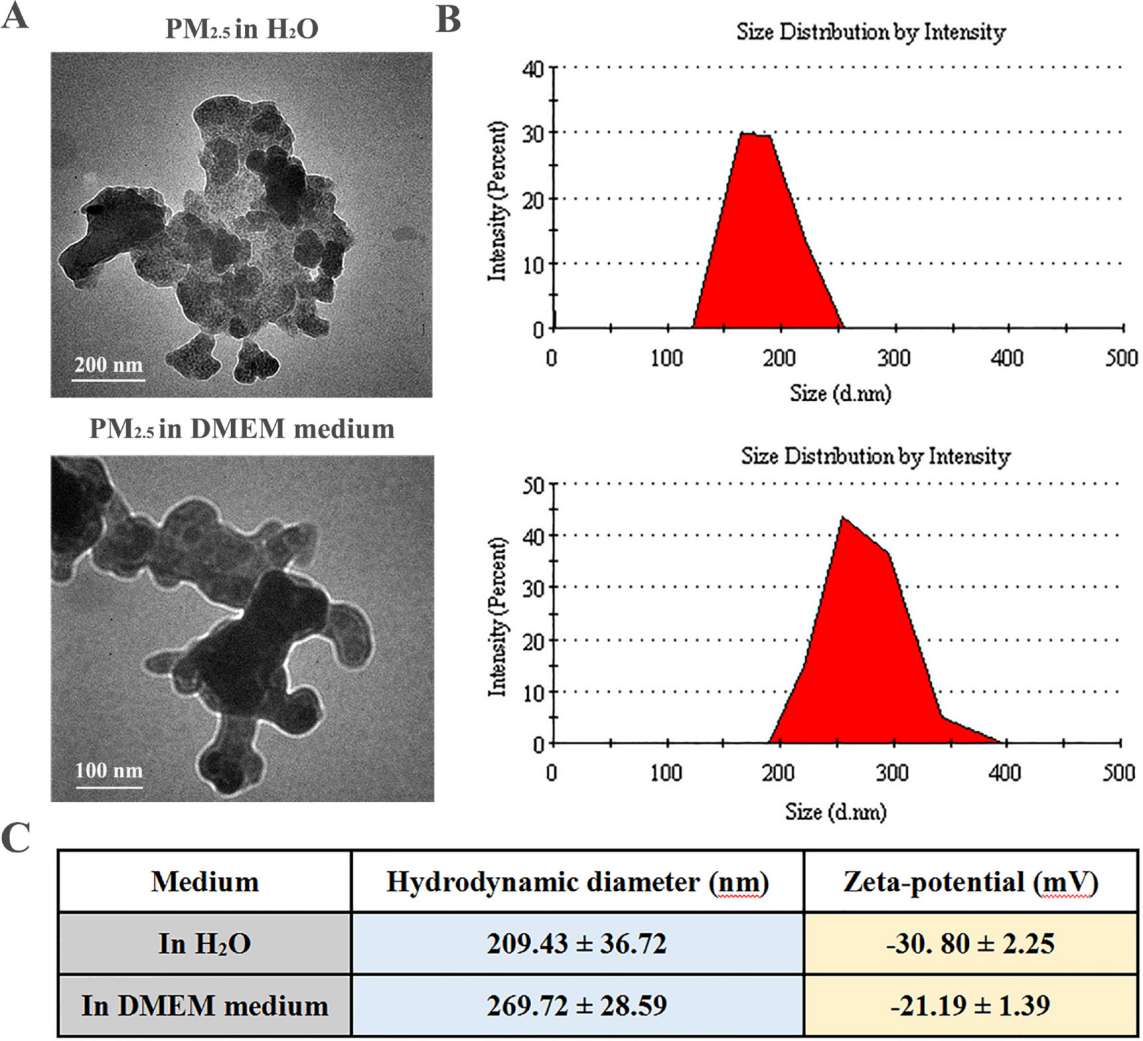
Morphological characterization of PM_2.5_ samples (**A**) Representative TEM images of PM_2.5_ in water and cell culture medium (magnification: 150 000× for the upper panel and 200 000× for the lower panel). (**B**) Gaussian fit curves of PM_2.5_ size distribution. (**C**) The hydrodynamic diameter and zeta potential of PM_2.5_ samples measured in water and cell culture medium at 100 μg/mL (*n* = 5).

The physicochemical properties of PM_2.5_ collected from Taiyuan were characterized. As shown in Figure 1A, transmission electron microscope (TEM) results revealed that PM_2.5_ appeared in irregular shapes in Milli-Q water or culture medium. The size distribution analysis showed around 30% of particles in PM_2.5_ ranged from 130 to 256 nm in water, and around 42% from 198 to 397 nm in DMEM medium (Figure 1B). The size of PM_2.5_ samples were confirmed by zeta-sizer measurement (Figure 1C). The zeta-potential data also indicated that PM_2.5_ exhibited strong negative charge in water. Of note, PM_2.5_ samples were negatively charged in cell culture medium with 10% FBS, likely due to the formation of protein corona on particle surface in biological settings [23].

In addition, the chemical characteristics of ambient PM_2.5_ during wintertime in Taiyuan, China, have been reported in our previous study [22]. Briefly, the investigated PM_2.5_ mass concentrations (0.161 ± 0.060 mg/m^3^), BaP equivalent toxicity (28.632 ng/m^3^) and individual carcinogenicity index (3.14 × 10^–5^) were much higher than those of the Chinese national recommended safety standards, indicating that PM_2.5_ pollution in Taiyuan might has carcinogenic potential to human health. The levels of PAHs, NPAHs and metals in PM_2.5_ were obviously higher than those of the Chinese national standard. The data from ion chromatography indicated that the daily mean levels of SO_4_^2−^ and NO_3_^−^ ions in the PM_2.5_ samples reached 5.87 and 1.71 μg/m^3^, respectively. The mean concentrations of Zn, Pb, As, Cd and Cu in PM_2.5_ samples were 0.76 ± 0.45, 0.30 ± 0.16, 0.025 ± 0.013, 0.0048 ± 0.0033, and 0.040 ± 0.025 μg/m^3^, respectively.

### Conditioned medium from PM_2.5_-treated 16HBE cells promotes the differentiation of BMSCs

**Figure 2:**
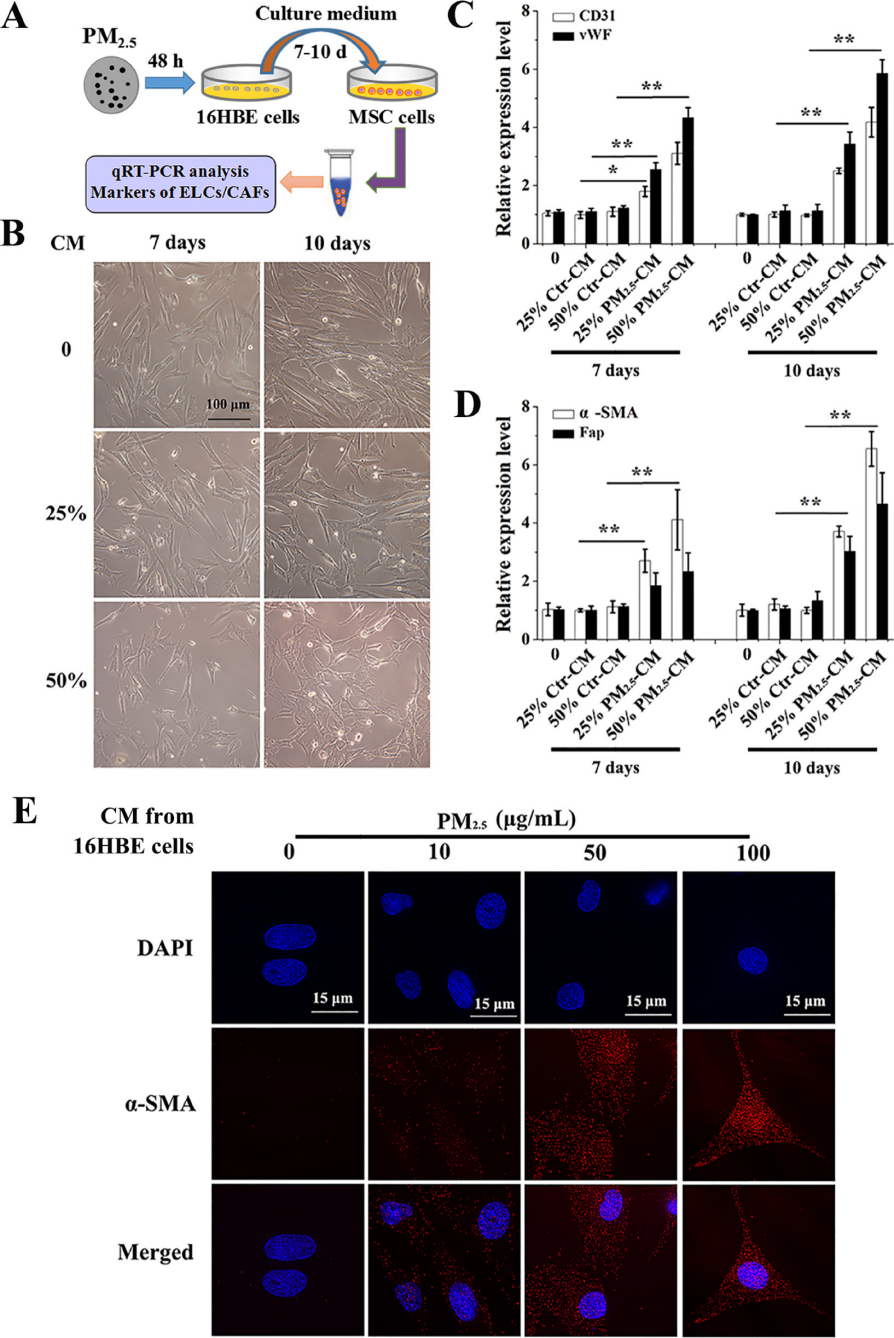
Conditioned medium from PM_2.5_-treated 16HBE cells induces the differentiation of BMSCs (**A**) Experimental protocol for PM_2.5_-exposed cells. 16HBE cells were treated with PM_2.5_ (10–100 μg/mL) for 48 hours, cell culture supernatants were removed, centrifuged and diluted 1:4 or 1:2 with DMEM/HBMSC-GM medium (without serum). BMSCs were exposed to CM from 16HBE cells for 7 or 10 days. (**B**) The morphology of BMSCs. mRNA levels of (**C**) CD31, vWF, (**D**) a-SMA and Fap were measured. (**E**) The localization of a-SMA in BMSCs upon 50% CM exposure for 7 days were determined (60**×** magnification); scale bars = 15 μm. The values were showed as means ± SD of triplicate determinations. ^*^p < 0.05, ^**^p < 0.01, compared with control.

To explore the influence of PM_2.5_-stimulated inflammatory secretions on BMSCs differentiation, we built an *in vitro* model. As shown in Figure 2A, a conditioned media (CM) approach was developed to analyze the role of PM_2.5_-stimulated human bronchial epithelial derived factors in the differentiation of human BMSCs (HBMSCs). The morphology of HBMSCs was studied by inverted microscope, and displayed flat and irregular in shape, similar to vascular endothelial cells (ECs), after exposure to 50% CM from 100 μg/mL PM_2.5_-treated human bronchial epithelial cells (16HBE cells) for 10 days, while the control group displayed long-spindle and vortex-like growth (Figure 2B). Strikingly, an increase in CD31 and vWF was observed after exposure to 25% and 50% CM from 100 μg/mL PM_2.5_-exposed 16HBE cells for 10 days (Figure 2C). Concomitantly, the levels of a-SMA and Fap were enhanced in response to 25% and 50% CM in 100 μg/mL PM_2.5_ groups, compared with counterpart from from control 16HBE cells for 7 or 10 days (Figure 2D). The immunofluorescence assay was performed to confirm the a-SMA expression, and the results illustrated an accumulation of a-SMA in nuclear upon 50% CM from 10, 50, 100 μg/mL PM_2.5_-treated 16HBE cells exposure for 7 days (Figure 2E). The above data showed that the respiratory secretions stimulated by PM_2.5_ promoted differentiation of BMSC into ELCs and CAFs.

### CM from PM_2.5_-treated 16HBE cells promotes the differentiation of BMSCs via the expressions and secretions of inflammatory cytokines

**Figure 3:**
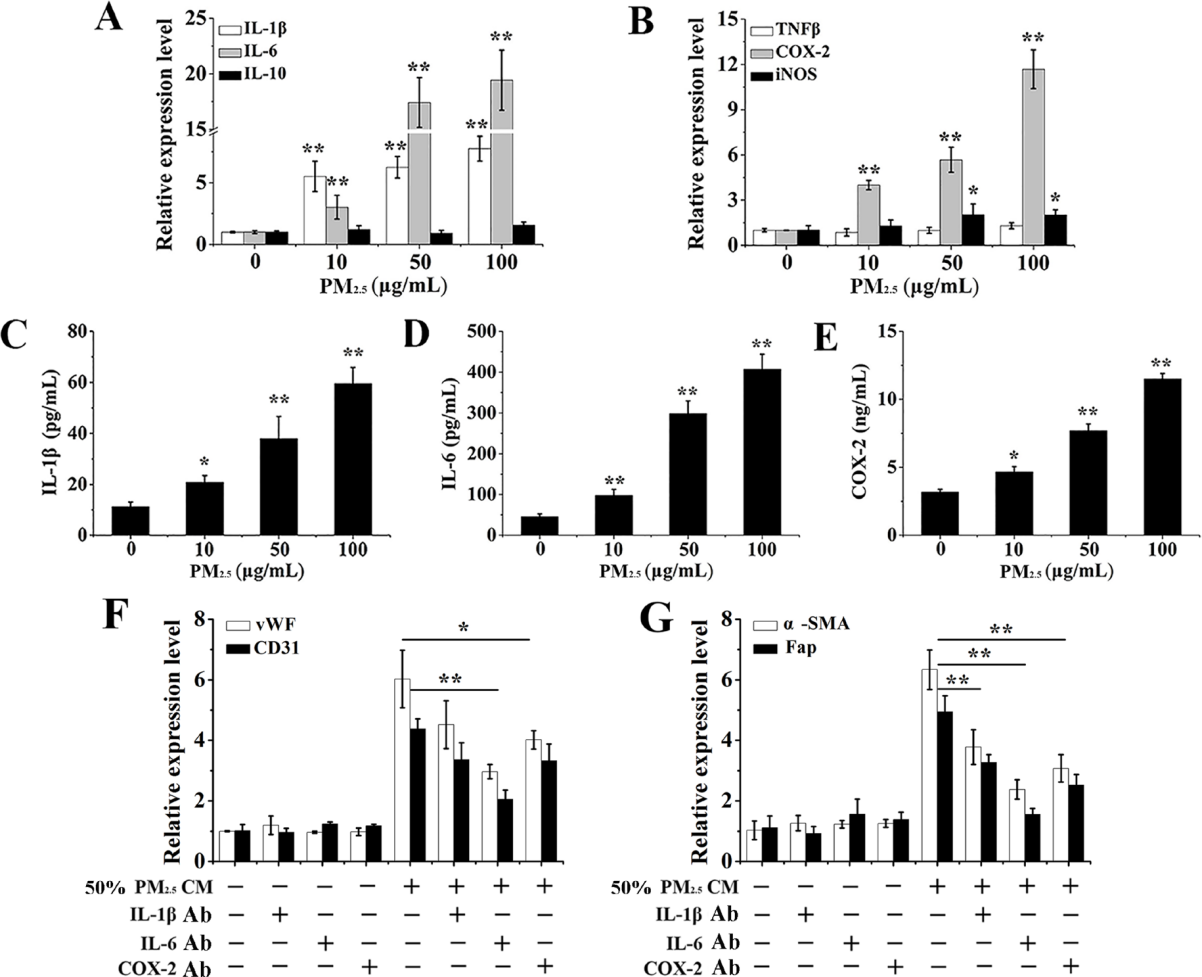
The effects of PM_2.5_-induced inflammatory cytokines from 16HBE cells on BMSCs differentiation mRNA levels of (**A**) IL-1β, IL-6, IL-10, (**B**) TNFβ, iNOS and COX-2 in 16HBE cells. (**C**–**E**) IL-1β, IL-6 and COX-2 secretions in cell culture supernatants of 16HBE cells. Then, conditioned medium from 100 μg/mL PM_2.5_-treated 16HBE cells was incubated with neutralization antibody against IL-1β (5 μg/mL), IL-6 (10 μg/mL), or COX-2 (2 μg/mL) at 37°C for 30 minutes before adding to the BMSCs. Gene expressions of (**F**) CD31, vWF, (**G**) a-SMA and Fap after 10 days of exposure were evaluated. Data represented were mean ± SD of three identical experiments. ^*^p < 0.05, ^**^p < 0.01, compared with control.

Inflammatory cytokines are an important secretion, produced by a PM_2.5_-stimulated respiratory tract [12, 13]. Moreover, extensive studies indicated that inflammatory cytokines had a critical role in the differentiation of BMSCs [24]. To explore whether inflammatory cytokines play an important role in the phenomenon, we then examine the effects of PM_2.5_ exposure on expressions of inflammatory cytokines in 16HBE cells by qRT-PCR assay. Our results showed significant increased expressions of IL-1β, IL-6 and COX-2 mRNA in a dose-dependent manner, no noticeably change in mRNA levels of IL-10 and TNFβ, and a slight increased iNOS expression in PM_2.5_-exposed 16HBE cells (Figure 3A and 3B). Specially, the relative mRNA expression of IL-1β, IL-6 and COX-2 were about 7.75 ± 1.08, 19.42 ± 2 .76 and 11.68 ± 2.28 upon exposure to 100 μg/mL PM_2.5_. In addition, an ELISA assay was carried out to determine the content of inflammatory mediators in CM from PM_2.5_-treated 16HBE cells. A prominent increase in the release of interleukin, including IL-1β and IL-6, in cell culture supernatants was observed upon 100 μg/mL PM_2.5_ exposure for 48 hours, while their levels trended upwards with the highest values in response to 100 μg/mL PM_2.5_ (Figure 3C and 3D). Moreover, PM_2.5_-treated cells secreted more COX-2 in a dose-dependent manner, when compared to control group (Figure 3E).

Furthermore, a cytokine neutralization assay was performed to investigate the effect of PM_2.5_-induced inflammatory cytokines on BMSCs differentiation. The role of IL-1β, IL-6 and COX-2 was established in the observed responses utilizing neutralizing antibodies against them. IL-6 played a significant role in the induction of tested differentiate markers (CD31, vWF, a-SMA, Fap) (Figure 3F and 3G). Interestingly, all cytokines, including IL-1β, IL-6 and COX-2, appeared to contribute to the induction of a-SMA and Fap as their neutralization afforded significant protection (Figure 3G). These results imply that inflammatory cytokines in CM from PM_2.5_-treated 16HBE cells are important for the differentiation of BMSCs.

### The differentiation of BMSCs provoked by inflammatory cytokines is mediated by PM_2.5_-induced ROS from NOX

**Figure 4:**
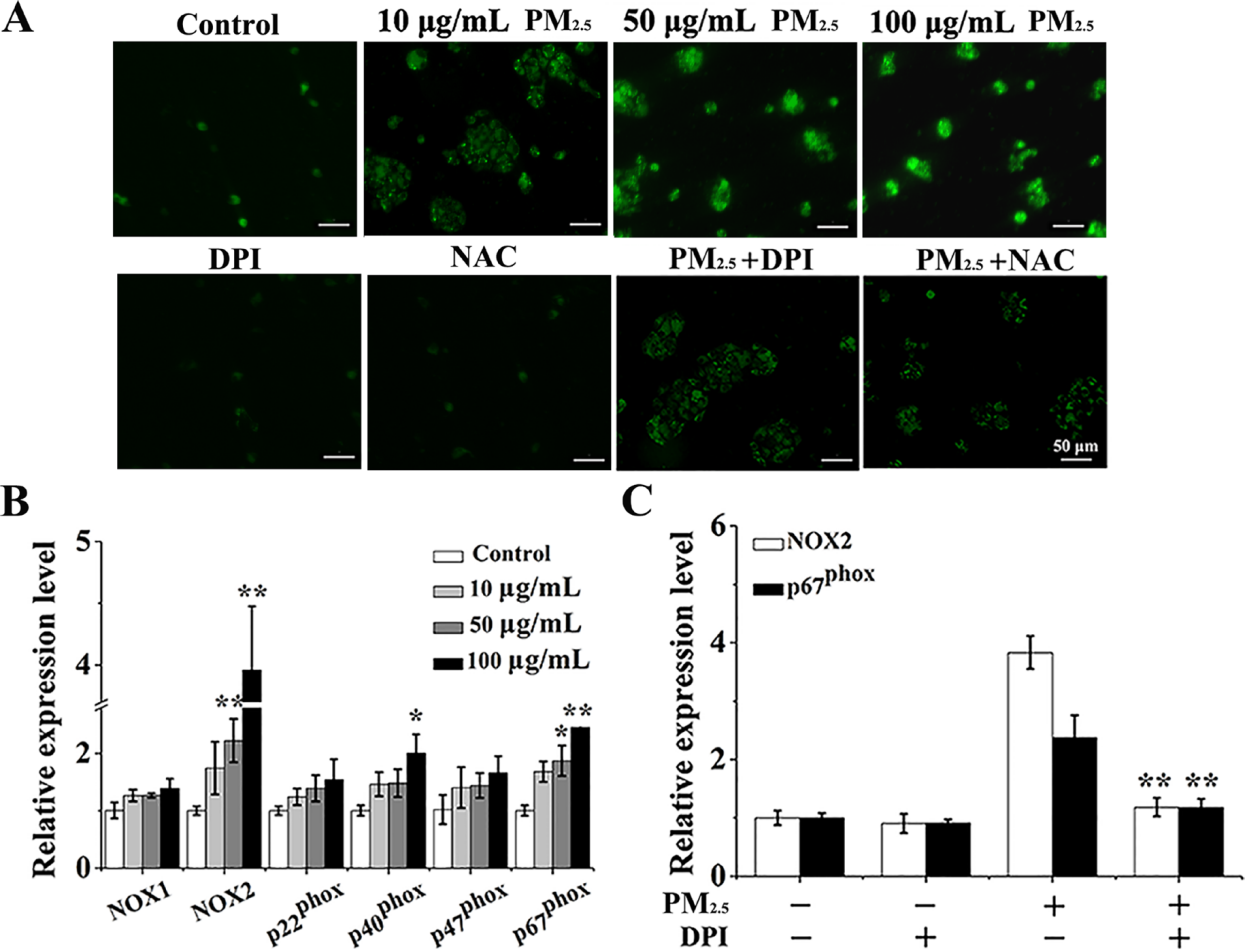
PM_2.5_ elevates the ROS production from NOX in 16HBE cells (**A**) After pre-treated with fresh media containing DPI (3 μΜ) or NAC (1 mM) for 6 hours and then treated with 100 μg/mL PM_2.5_ in the presence of inhibitors for 48 hours, representative microphotographs showing ROS content in 16HBE cells. Images were captured by a fluorescence microscope (20× magnification), scale bars = 50 μm. (**B**) qRT-PCR assay was carried out to measure the expressions of NOX subunits. (**C**) After pre-treated with fresh media containing DPI (3 μΜ) for 6 hours and then treated with 100 μg/mL PM_2.5_ suspensions in the presence of inhibitors for 48 hours, the expressions of p22^phox^ and p67^phox^ were measured. Data represented were mean ± SD of three identical experiments. Statistically significant different from control: ^*^p < 0.05, ^**^p < 0.01.

Given that ROS was accountable for the inflammatory response [16] and was the main target of PM_2.5_-caused toxicity on human health [17], we next focused on ROS generation and studied the mechanism of PM_2.5_-activated inflammation on BMSCs differentiation. Microscopic fluorescence imaging revealed that PM_2.5_ induced a significant dose-dependent production of ROS (Figure 4A). To explore the source of PM_2.5_-elevated ROS, the expressions of NADPH oxidase (NOX) subunits were examined, which are a key source of ROS generation [25]. The assay of qRT-PCR revealed that PM_2.5_ treatment resulted in apparently elevated mRNA levels of NOX2 and p67^phox^ (Figure 4B). In order to further confirm the possible role of NOX in ROS generation, DPI, the inhibitor of ROS from NOX family, and NAC, the normal inhibitor of ROS, were applied. mRNA levels of NOX2 and p67^phox^ elevated by PM_2.5_ were rescued by the DPI addition (Figure 4C). Meanwhile, after pre-treatment with DPI or NAC for 24 hours, ROS levels were reduced compared to PM_2.5_ treatment alone (Figure 4A). Of note, this reversed effect of NAC was more prominent than DPI, indicating that ROS induced by PM_2.5_ could generate from other sources apart from NOX.

**Figure 5:**
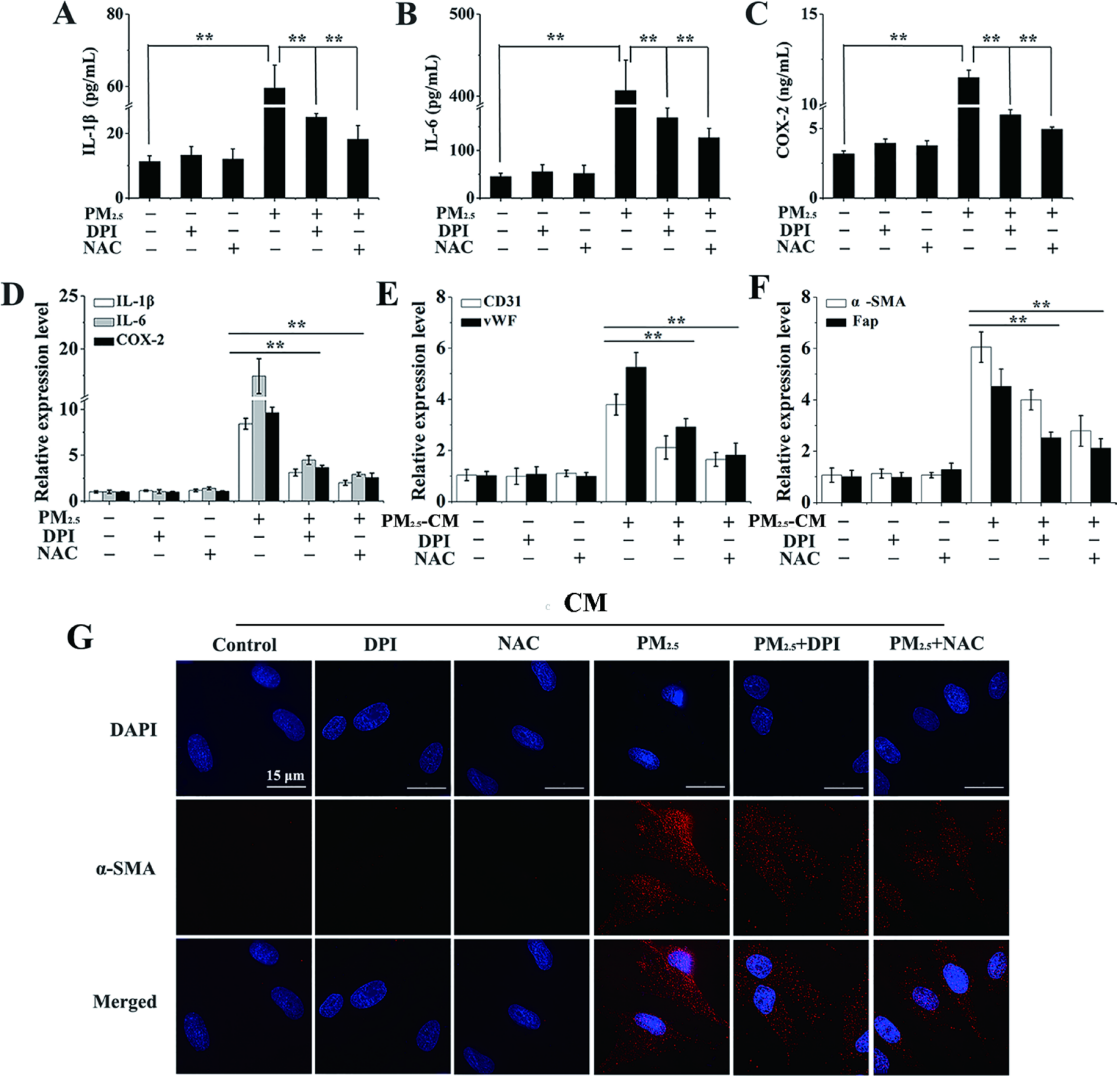
The differentiation of BMSCs provoked by inflammatory cytokines is mediated by PM_2.5_-induced ROS generation (**A**–**C**) After 16HBE cells were treated with PM_2.5_ for 48 hours with or without the pre-treatment of DPI (3 μΜ) or NAC (1 mM) for 6 hours, ELISA was performed to investigate inflammatory cytokines secretions. (**D**) Expressions of inflammatory cytokines were also measured in 16HBE cells. The expressions of (**E**) CD31, vWF, (**F**) a-SMA and Fap in BMSCs were observed when secretions from 16HBE cells pre-treated with DPI or NAC were used. (**G**) The localization of a-SMA in BMSCs (60**×** magnification), scale bars = 50 μm. Error bars indicated the means ± SD of triplicate determinations. ^*^p < 0.05, ^**^p < 0.01, compared to PM_2.5_-treated cells.

To further investigate whether CM triggered differentiation of BMSCs was mediated by epithelial derived inflammation factors, CM with or without DPI or NAC pre-treatment was added. Firstly, the secretions and expressions of inflammatory cytokines were studied by ELISA and qRT-PCR assay. Notably, a decrease in the elevated release of inflammatory mediators (IL-1β, IL-6 and COX-2) in PM_2.5_-treated cell culture supernatants was observed upon DPI or NAC supplement (Figure 5A–5C). Consistently, IL-1β, IL-6 and COX-2 gene expressions were also significantly reduced by the addition of DPI or NAC (Figure 5D). To further investigate whether CM triggered differentiation of BMSCs was mediated by epithelial derived inflammation factors, CM with or without DPI or NAC pre-treatment was added. Interestingly, a significant decrease in mRNA levels of CD31, vWF, a-SMA and Fap was observed when CM from 16HBE cells pre-treated with DPI or NAC was used (Figure 5E and 5F). This consistent effect was confirmed by the prominently repressed of a-SMA nuclear translocation in BMSCs in response to CM with DPI or NAC pre-treatment (Figure 5G). It therefore concludes that the differentiation of BMSCs into CAFs and ELCs is dependent on epithelial cells-derived inflammatory cytokines mediated by PM_2.5_-induced ROS from NOX.

### The differentiation of BMSCs caused by PM_2.5_-induced inflammatory cytokines is mediated by ROS derived from mitochondria

**Figure 6:**
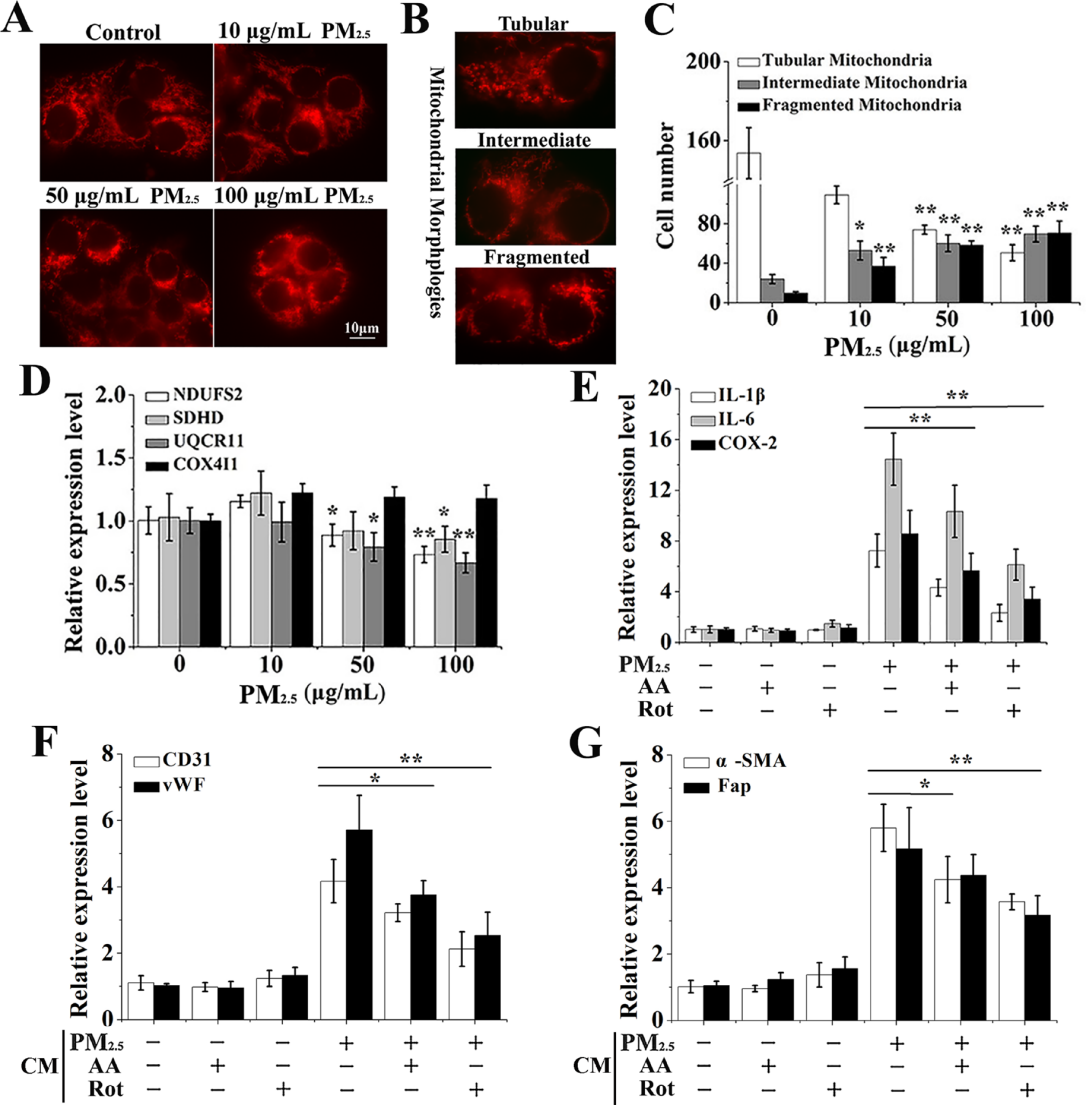
The differentiation of BMSCs provoked by inflammatory cytokines is mediated by PM_2.5_-induced ROS from mitochondria (**A**) MitoTracker Red was carried out to assess the PM_2.5_-induced alteration in mitochondrial morphology. (**B**) Different types of typical mitochondrial morphologies. (**C**) Quantitative analysis of alteration of mitochondrial morphology in PM_2.5_-treated cells. (**D**) The complexes of mitochondrial respiratory chain, including NDUFS2, SDHD, UQCRI1 and COX4I1, were investigated by qRT-PCR. After 16HBE cells were treated with PM_2.5_ for 48 hours with or without the pre-treatment of 5 μM Rot (inhibitor of mitochondrial complex I) or 2 μM AA (inhibitor of mitochondrial complex III) for 6 hours, (**E**) qRT-PCR was performed to investigate expressions of inflammatory cytokines in 16HBE cells. The expressions of (**F**) CD31, vWF, (**G**) a-SMA and Fap in BMSCs were observed when secretions from 16HBE cells pre-treated with Rot or AA. Data represented were mean ± SD of at least three identical experiments. ^*^p < 0.05, ^**^p < 0.01, compared to control.

Mitochondria is the other main source of ROS and direct stress organelles upon foreign pollutants exposure [26]. To verify if PM_2.5_-induced ROS was generated from other sources apart from NOX, we carried out a comprehensive evaluation of the impact of PM_2.5_ on mitochondrial structure and function. To assess the PM_2.5_-induced alteration in mitochondrial morphology, we used the MitoTracker Red, a fluorescence dye that stains mitochondria, and observed pronounced disorders of mitochondrial morphology in PM_2.5_-exposed cells (Figure 6A). When different types of mitochondrial morphologies were scored, we found that PM_2.5_ damaged the mitochondrial network. PM_2.5_-treated cells showed a diminished number of cells presenting preserved tubular mitochondria and an increased number of cells with fragmented mitochondria compared to control cells (Figure 6B and 6C). When mitochondria are damaged by pollutants, ROS is generated form the mitochondrial respiratory chain [26]. Therefore, four compound genes from the mitochondrial respiratory chain NDUFS2, SDHD, UQCRI1 and COX4I1, were further determined, and UQCRI1 and NDUFS2 levels were significantly inhibited by PM_2.5_ exposure (Figure 6D).

The role of ROS from mitochondria in PM_2.5_-stimulated inflammatory secretions and BMSCs differentiation was further investigated. Our results indicated that PM_2.5_-induced expression of inflammatory cytokines in 16HBE cells were notably inhibited by Rot (inhibitor of mitochondrial complex I) or AA (inhibitor of mitochondrial complex III), while inhibitor treatment alone showed no obvious effect (Figure 6E). CD31 and vWF expressions induced by PM_2.5_ in combination with AA or Rot treatment were about 3.21, 2.12, 3.75 and 2.53 folds with respect to control cells, respectively, which were statistically reduced relative to PM_2.5_ group (Figure 6F). Likewise, co-treatment with AA or Rot statistically inhibited a-SMA and Fap expression in contrast with PM_2.5_ treatment alone (Figure 6G). In summary, ROS derived from NADPH oxidase and mitochondria contributed to PM_2.5_-induced inflammatory secretions and BMSCs differentiation.

### PM_2.5_ exposure induces the BMSCs differentiation and inflammatory cytokines expression in SD rats

**Figure 7:**
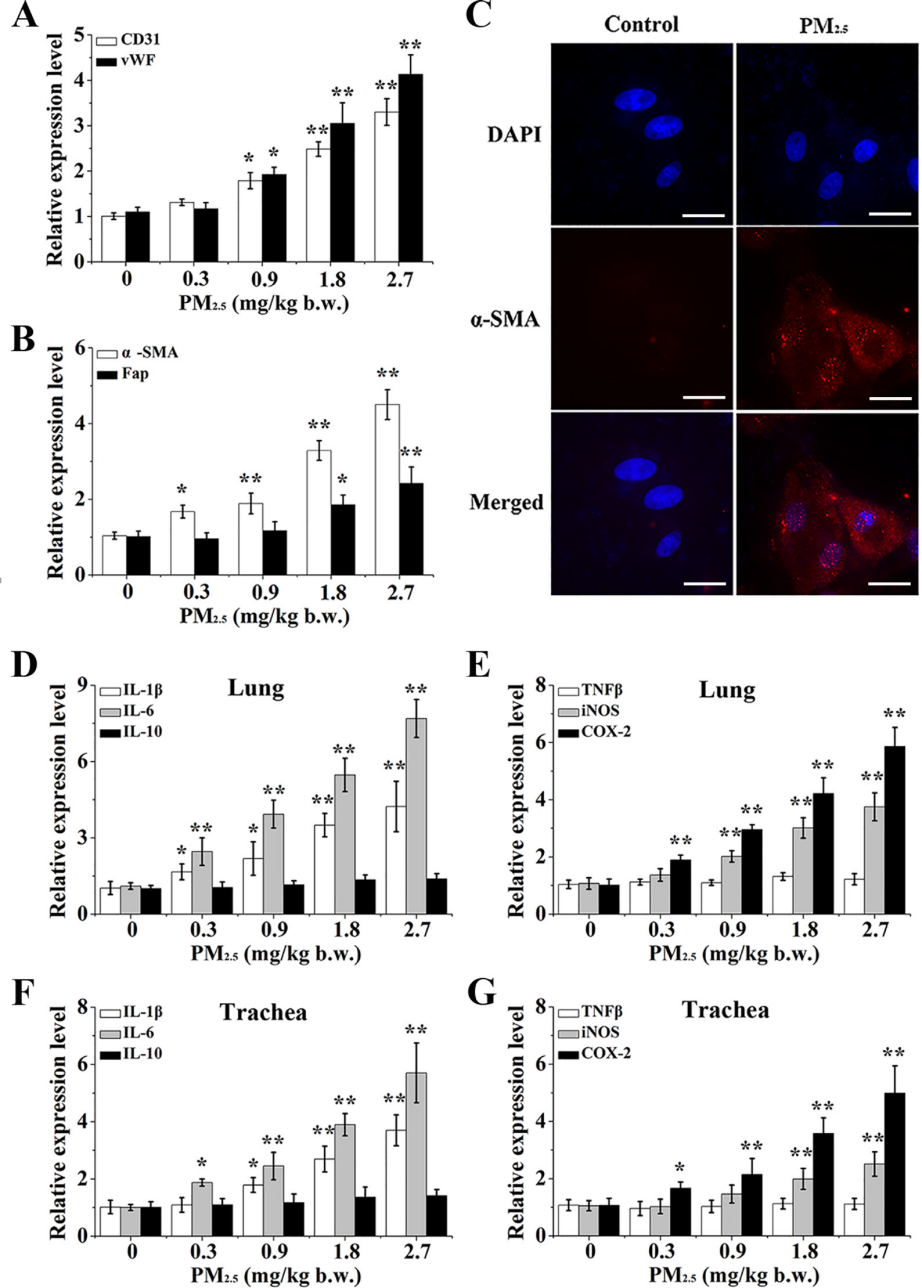
The differentiation of BMSC and inflammatory response caused by PM_2.5_ *in vivo* Gene expressions of (**A**) CD31, vWF, (**B**) a-SMA and Fap were observed in BMSCs. (**C**) The localization of a-SMA in BMSCs of 2.7 mg/kg b.w. PM_2.5_ exposed rats (60**×** magnification). Inflammatory cytokines, including (**D**, **F**) IL-1β, IL-6, IL-10, (**E**, **G**) TNFβ, iNOS and COX-2 were determined in lung tissues or trachea tissues. Values shown were given as the mean ± SD of 6 animals in each group. ^*^p < 0.05, ^**^p < 0.01, compared with control mice.

The above results uncovered that PM_2.5_ enhanced the differentiation of BMSCs into ELCs and CAFs via the stimulation of inflammatory cytokines, which were mediated by ROS from NOX and mitochondria in human bronchial epithelial cells. To validate our findings, an *in vivo* assay in SD rat was performed to further determine the toxicity induced by PM_2.5_. After 0.3, 0.9, 1.8, 2.7 mg/kg body weight (b.w.) PM_2.5_ exposed to SD rats for 30 days with intratracheal instillation, we separated and extracted the BMSCs cells from the bone marrow of SD rats, and determined the differentiation of BMSCs into ELCs and CAFs (Supplementary Figure 1A). Interestingly, PM_2.5_ made a dose-related alteration on the morphology of BMSCs (Supplementary Figure 1B). The qRT-PCR result showed that levels of specific surface markers of ELCs, including CD31 and vWF (Figure 7A), as well as a-SMA and Fap, the reliable markers of CAFs (Figure 7B), in BMSCs were dose-dependently enhanced in response to PM_2.5_ exposure. Concomitantly, the consistent up-regulated expression of a-SMA was further confirmed by immunofluoresent staining, in which a-SMA in 2.7 mg/kg b.w. PM_2.5_ exposed-BMSCs localization was accumulated (Figure 7C).

Next, we measured effects of inflammatory cytokines in lung and bronchial tissues in response to PM_2.5_, and found PM_2.5_ significantly elevated the mRNA levels of IL-1β, IL-6 and COX-2 in a dose dependent manner in lung tissues (Figure 7D and 7E). Histological examination also revealed that 2.7 mg/kg b.w. PM_2.5_-treated tissues had a histological appearance with a pro-inflammation response, as indexed by the accumulation of IL-1β, COX-2 and NF-κB (Supplementary Figure 2A). The results coincided with those of the bronchial tissues (Figure 7F and 7G, Supplementary Figure 2B), indicating that PM_2.5_ activated the inflammatory response of the respiratory tract in SD rats.

## DISCUSSION

In the present study, we mechanistically investigated PM_2.5_-activated inflammation and elaborated its role in the induction of BMSCs differentiation using both *in vitro* and *in vivo* models. The data implicated that PM_2.5_ exposure elevated the ROS generation via binding to cell membrane-bound NOX and causing mitochondrial disorder, which further provoked the inflammatory cytokines expression and secretion in the respiratory tract. Moreover, the secretion promoted the differentiation of BMSCs into ELCs and CAFs (Supplementary Figure 3).

To exclude if all distractions of cell culture supernatants removed from 16HBE cells contain the chemicals that dissolved from the PM_2.5_, a negative control that contained DMEM and PM_2.5_ has been centrifuged, diluted, and added to the BMSCs to minimalize this influence. 50% CM from PM_2.5_-treated 16HBE cells substantially increased the expressions of markers of ELCs and CAFs, compared to the control group (HBMSC-GM medium), while there was no obvious alteration in negative control and 50% CM from 16HBE cells (Supplementary Figure 4). In our previous study, we have compared the effect of suspension from the blank filter and physiological saline on levels of SOD, iNOS, MDA, Na^+^K^+^-ATPase and Ca^2+^-ATPase of hearts in rats, and no statistical difference was observed between the normal control and suspension from the blank filter group, indicating that filter-derived quartz debris did not cause bias in our experimental outcomes [27].

To clarify whether the differentiation of BMSCs induced by PM_2.5_ was through inflammation, conditioned medium from PM_2.5_-treated 16HBE cells was extracted to treat the BMSCs. Our data depicted that inflammatory cytokines secreted from PM_2.5_-exposed 16HBE cells promoted the differentiation of BMSCs into ELCs and CAFs. This is an actual exposure method, which is more realistic and practical than the impact of direct exposure of PM_2.5_ on BMSC differentiation. As we all known, acute differentiation of BMSCs promotes tissue repair and regeneration, while its long-term and chronic differentiation upon persistent stimulation leads to over-repair, and a sharp increase of endothelial cells and smooth muscle cells in the blood vessels, causing the pathogenesis of diseases such as cancer, retinopathy, and atherosclerosis [28, 29]. Furthermore, PM was designated as a Group I carcinogen by the International Agency for Research on Cancer (IARC) of the World Health Organization (WHO) in 2013, and closely associated with increased cancer incidence, especially lung cancer [30]. Of note, there has been growing attention that BMSCs can travel to tumor stroma, where they differentiate into ELCs and CAFs, which may lead to the development of cancer [6, 31]. Accordingly, our data may provide one possible mechanism of PM_2.5_-induced cancers.

Many inflammatory mediators are found in the tumor microenvironment and participate in the directed migration of stem cells, such as BMSCs, to tumors and inflamed microenvironments [32]. Cancer progression has been correlated with an increase in inflammatory mediator gene expression, which is thought to occur via disruption, damage and cellular turnover occurring in the tumor microenvironment. Constant production of inflammatory mediators perpetuates the maintenance and progression of the tumor environment, which becomes a target for the BMSCs [31]. The stimulation of BMSCs by inflammatory cytokines in the tumor microenvironment enhanced tumor angiogenesis, leading to cancer growth in mice model [33]. In the present study, we indicated that PM_2.5_ exposure may stimulate the inflammation-associated cytokine activation. Consistently, numerous studies have revealed that inflammation responses were the direct and primary response upon PM_2.5_ exposure [34]. For example, long-term PM_2.5_ exposure increased mouse blood pressure that involved hypothalamic inflammation [35]. Particle pollution in Rio de Janeiro, Brazil increased pro-inflammatory IL-6 in human lung cells [36].

Several reports suggest ROS is an important molecular mechanism of PM_2.5_-mediated toxicity [37, 38]. Our data also showed that PM_2.5_-induced ROS might be involved in the activation of inflammation. Evidence indicated that ROS was dedicated to regulating inflammatory gene transcription [39]. Importantly, oxidative stress, induced by PM_2.5_, has been reported to increase the production of mediators of pulmonary inflammation and initiate or promote mechanisms of carcinogenesis [40]. Our results indicated that PM_2.5_ enhanced the ROS generation from either promoted NOX or mitochondria. The NOX system is a dominating source of ROS and its activation is correlated with membrane-bound proteins (Nox2 and p22phox) and regulatory cytosolic components (p40^phox^, p47^phox^, and p67^phox^) [25]. During oxidative phosphorylation, electrons are moved thorough the mitochondrial respiratory chain, and a proton gradient is established across the inner mitochondrial membrane as the energy source for ATP production [41]. When mitochondria are damaged by pollutants, ROS is generated from the mitochondrial respiratory chain [26]. There are many reports that the PM exposure induced the ROS generation by affecting NOX expressions or mitochondria disorders [19, 42], but they did not fully compare the contribution of NOX and mitochondria on the PM_2.5_-caused ROS generation. The present results indicated PM_2.5_ exposure resulted in higher ROS generation in 16 HBE cells, through promoted NAPDH oxidase activation and mitochondrial respiratory chain activation.

The selection basis of dosage of PM_2.5_ in our study can be listed as follow. As has been reported, the respiratory volume of an adult rat was 200 mL/min, and the respiratory volume for 3 days reached 0.864 m^3^. According to the China National Ambient Quality Standard (NAAQS, 2012) for PM_2.5_ (0.075 mg/m^3^), the amount of PM_2.5_ inhalation over 3 days is 0.0648 mg and the concentration of PM_2.5_ exposure for each rat every 3 days is estimated to be 0.324 mg/kg b.w. (rat 200 ± 20 g). PM_2.5_ mean mass concentration determined in our samples was 0.161 ± 0.060 mg/m^3^ on non-haze weather in Taiyuan [22], and the higher concentrations corresponded to the haze weather reached 0.692 ± 0.272 mg/m^3^ [43]. Taken together, the average concentrations of PM_2.5_ exposure for each rat every 3 days were estimated in the range from 0.324 to 2.989 mg/kg b.w.. Therefore, we selected 0.3, 0.9, 1.8, 2.7 mg/kg b.w. PM_2.5_ as exposure doses in our SD rat experiment.

To the best of our knowledge, this is first report to examine the detrimental effect of PM_2.5_-stimulated inflammatory cytokines secreted from respiratory tract on stem cell differentiation. Besides, we analyze the sources of PM_2.5_-caused ROS generation from NADPH oxidases (NOXs) and mitochondria. Accordingly, this project reveals a mechanistic and theoretical basis for stem cells toxicology induced by PM_2.5_, which reflects the association of particle pollution with risks for human health, and assists the development of preventive treatments for urban dust-haze.

## MATERIALS AND METHODS

### PM_2.5_ preparation and physiochemical characterization

PM_2.5_ samples were collected during winter 2012/2013 in Taiyuan, a site of Shanxi urban background for atmospheric pollution. As described in our previous study [22], the sampling sites were located in Taiyuan, Shanxi province, China (30°15′N latitude, 112°33′E longitude). A PM_2.5_ high volume air sampler (Thermon Anderson, USA) was placed on the rooftop of a building about 25 meters tall, with no any obstacles and no large pollution sources near the building. PM_2.5_ concentrations were measured using a DustTrakTM II Aerosol Monitor (TSI Inc., USA), and daily PM_2.5_ samples were collected on quartz fiber filters (QFFs) with a pump flow rate of 1.13 m^3^/min. These filters loading samples were cut and surged in Milli-Q water with sonication. To obtain PM_2.5_ suspensions, the above suspensions were freeze-dried under vacuum and weighed. The samples were then dried and stored at –20°C. The 20 mg PM_2.5_ particles supplemented with 1 mL 0.9% physiological saline were blended, treated with ultrasonic oscillation for 30 min. Particles were UV-irradiated overnight to inactivate possible contaminating endotoxin and to be germicidal, as indicated in the paper of Paul M Peeters *et al.* [44]. Then 20 mg/mL PM_2.5_ was stored at 4°C, with gentle oscillation before use. Prior to use, PM_2.5_ suspensions were diluted with sterilized 0.9% physiological saline, surged for 20 min and mixed fully.

The PM_2.5_ morphology was examined using transmission electron microscopy (TEM) (Hitachi, H7650, Japan), and diameter distributions were calculated based on the TEM analysis. The hydrodynamic diameter and surface charge of PM_2.5_, either suspended in Milli-Q water or in culture medium, was measured by a zeta-sizer (Malvern Nano ZS, Nalvem, UK) using a standard method [45]. The concentrations of polycyclic aromatic hydrocarbons (PAHs) in PM_2.5_ during sampling period were measured by gaschromatography–mass spectrometer (GC-MS), and the concentrations of nitrate (NO_3_^−^) and sulfate (SO_4_^2–^) were analyzed by ion chromatography.

### Cells and culture conditions

Human bone marrow mesenchymal stem cells (HBMSCs) were purchased from Chinese Biowit Technologies and cultured in HBMSC-GM (Biowit, China). The 16HBE cell line (human bronchial epithelial cells), obtained from the Institute of Biochemistry and Cell Biology (SIBS, CAS, Shanghai, China), was maintained in DMEM medium (HyClone) supplemented with 10% FBS (Boster) and 1% penicillin/streptomycin (Solarbio). Cells were kept at 37°C in a 5% CO_2_ humidified cell culture incubator. 16HBE cells were treated with different doses of PM_2.5_ (0, 10, 50 and 100 μg/mL) for 48 hours, cell culture supernatants were removed, centrifuged (2000 rpm, 4°C, 15 min) and diluted 1:4 (25%) or 1:2 (50%) with DMEM/HBMSC-GM culture medium (CM) without serum. BMSCs were exposed to 25% or 50% CM for 7 or 10 days. All inhibitors were obtained from Sigma-Aldrich (St. Louis, MO). 16HBE cells were pre-treated for 6 hours with one of the following enzyme inhibitors: 3 μΜ diphenyleneiodonium chloride (DPI, inhibitor of NADPH oxidase); 1 mM N-acetyl-L-cysteine (NAC, inhibitor of ROS); 5 μM Rotenone (Rot, inhibitor of mitochondrial complex I) or 2 μM Antimycin A (AA, inhibitor of mitochondrial complex III). After inhibitor pretreatment, cells were incubated another 48 hours with 100 μg/mL PM_2.5_ suspensions. Experiments were repeated at three separate times in duplicates. For cytokine neutralization assays, cells were incubated for 30 minutes with neutralizing antibodies (anti IL-1β, anti IL-6 and anti COX-2 (MBL, Japan) before treatment with PM_2.5_ (in the presence of antibodies) for an additional 48 hours.

### Quantitative real time PCR

Quantitative real time PCR (qRT-PCR) was employed using an Applied Bio-systems platform, according to our previous methods [46]. Human or rat primers for targeted or reference genes were shown in Supplementary Tables 1 and 2, respectively.

### Immune-fluorescence assay

To perform the immune-fluorescence assay, cells were grown on 12-well glass slides. After experimental procedures, cells were washed with PBS, fixed with 4% paraformaldehyde and permeated in PBS containing 0.1% Triton. Afterwards, cells were blocked with 3% BSA in PBS and incubated for 1 hour with a-SMA (1/500) primary antibody (Biotime, China). The slides were then washed and incubated with corresponding anti-rabbit-TRITC secondary antibody (1/100) (Proteintech, China). The nucleus was then stained with DAPI for 30 min. After washing with PBS, the slides were mounted in gelvatol for confocal immune-fluorescence analysis. Images were acquired with a fluorescence microscope (Delta Vasion, USA) at 60× magnification.

### Cytokine analysis

The concentrations of IL-1β, IL-6 and COX-2 released into the culture supernatant after different doses PM_2.5_ exposure with or without NAC or DPI treatment were evaluated using commercially available human enzyme-linked immunosorbent assay (ELISA) kit (Westang, Shanghai), according to manufacturer's recommendations.

### Determination of ROS generation

Generation of ROS was determined by microscopic fluorescence imaging using 2,7-dichlorofluorescin diacetate (DCFH-DA), which was obtained from Beyotime Institute of Biotechnology (Nan tong, China) as described by our previous methods [42].

### Analysis of mitochondrial morphology

16HBE cells were incubated with the cell-permeant mitochondria-specific red fluorescent probe MitoTracker Red CMXRos at a final concentration of 100 nM in serum free-culture medium at 37°C for 30 min. Afterwards, cells were washed twice with PBS and fixed with 4% paraformaldehyde (20 min at RT). Finally, cells were mounted on glass slides, and samples were examined under a fluorescence microscope (Delta Vasion, USA) (60×). To quantify the different mitochondrial morphologies, 200 cells/sample were scored and classified as cells exhibiting tubular (normal), intermediate (tubular with swollen regions) and fragmented (small and globular) mitochondria according to the paper of Alaimo *et al.* [47].

### Animal experiment

As indicated in our previous study [27], healthy adult, clean-grade male Sprague-Dawley (SD) rats (weighing 200 ± 20 g) were commercially obtained from the Experimental Animal Center of the Chinese Military Medical Science Academy (Beijing, China). The animals were kept in standard animal houses under a 12 h light/dark cycle (lights on at 8:00 a.m.) at a constant temperature of 24 ± 2°C and relative humidity of 50 ± 5%. After 7 days of habituation, the rats were randomly divided into the following five groups (*n* = 6), (1) control group, (2) 0.3 mg/kg body weight (b.w.) PM_2.5_ group, (3) 0.9 mg/kg b.w. PM_2.5_ group, (4) 1.8 mg/kg b.w. PM_2.5_ group and (5) 2.7 mg/kg b.w. PM_2.5_ group. The treatment groups were instilled with a 0.5 mL PM_2.5_ suspension, and the final exposure concentrations reached 0.3, 0.9, 1.8, 2.7 mg/kg b.w., while the control group was instilled with physiological saline at the same volume as that was used for the treatment group. The instillation was conducted using a nonsurgical intratracheal instillation method [48], and performed once every 3 days. When not being treated, the rats had free access to food and water. All of these rats were maintained under standard nutritional and environmental conditions throughout the experiment according to the requirement of the National Act on the Use of Experimental Animals (China). Maximal effort was made to minimize animal suffering and the number of animals necessary for the acquisition of reliable data.

### The isolation and culture of murine bone marrow mesenchymal stem cells

Murine bone marrow mesenchymal stem cells (BMMSCs) were isolated from femurs and tibias of SD rat, according to our previous methods [49]. Cells were seeded in culture dishes with complete medium constituted of IMDM (Gibco, Grand Island, NY, USA), 10% fetal bovine serum (Gibico), and penicillin/streptomycin (50 U/ml and 50 mg/ml, respectively; Gibco-Invitrogen, Carlsbad, USA). Cultures were incubated at 37°C with 5% fully humidified CO_2_. After 24 hours, non-adherent cells were removed by washing with 1× PBS, and fresh medium was added. The monolayer cells were then used to qRT-PCR and immune-fluorescence assay.

### Statistical analysis

Statistical analysis was carried out using the SPSS 17.0 software program. Data, derived from at least three independent experiments, were presented as the mean ± SD, and analyzed by one-way analysis of variance (ANOVA) followed by Tukey's post hoc test. A value of ^*^p < 0.05 and ^**^p < 0.01 was considered statistically significant.

## SUPPLEMENTARY MATERIALS FIGURES AND TABLES


