# Supplementary material for: Crucial role of pro-inflammatory cytokines from respiratory tract upon PM2.5 exposure in causing the BMSCs differentiation in cells and animals
Source: Oncotarget. 2017 Dec 11;9(2):1745–59. doi: 10.18632/oncotarget.23158 (PMC5788596; doi:10.18632/oncotarget.23158)
Supplement: Supplementary file 1 [file oncotarget-09-1745-s001.pdf]

Crucial role of pro-inflammatory cytokines from respiratory tract upon $PM_{2.5}$ exposure in causing the BMSCs differentiation in cells and animals

SUPPLEMENTARY MATERIALS

Immuno-histochemical analysis

Rats were killed by anesthesia (sodium pentobarbital, 80 mg/kg, i.p.) 24 hours after the last treatment. Lung and bronchial tissues were immediately removed, washed in sterile 0.9% (w/v) sodium chloride solution and cut into two parts. One part was immediately fixed in 10% formalin over 24 hours, dehydrated and then embedded in paraffin wax. The section was used for histopathological examination and stained with antibodies to IL-1β, COX-2 and NF-κB. After washing, they were stained with horseradish peroxidase-conjugated secondary antibody (ZSGB-BIO, China), incubated with streptavidin-horseradish peroxidase complex, stained with diaminobenzidine (DAB), and then counterstained with hematoxylin. The remaining part was collected in RNasefree tubes and snapped frozen in liquid nitrogen. The frozen tissues were stored at –80°C until these tissues were used for RNA extraction.

Supplementary Table 1: Primers of human used for quantitative real-time polymerase chain reaction (qRT-PCR)

Gene	Primers	Sequences (5′→3′)
CD31	Forward	AGACGTGCAGTACACGGAAG
	Reverse	AGTATCTGCTTTCCACGGCA
vWF	Forward	GGCGTTATAACAGCTGTGCG
	Reverse	GACAGTGTGCAGGGTCATCA
a-SMA	Forward	AGCGTGGCTATTCCTTCGT
	Reverse	CCATCAGGCAACTCGTAACTC
IL-1β	Forward	TTACAGTGGCAATGAGGATG
	Reverse	TGTAGTGGTGGTCGGAGATT
IL-6	Forward	CCTTCGGTCCAGTTGCCTTCT
	Reverse	CAGTGCCTCTTTGCTGCTTTC
IL-10	Forward	AGAACCAAGACCCAGACATCA
	Reverse	GCATTCTTCACCTGCTCCAC
TNFβ1	Forward	CGAGTGACAAGCCTGTAGCC
	Reverse	TGAAGAGGACCTGGGAGTAGAT
iNOS	Forward	CAGGACTCACAGCCTTTGGA
	Reverse	CTGGATGTCGGACTTTGTAGATT
COX-2	Forward	GTCTAAATCGGGAGTTGGAATCA
	Reverse	CACAGTATGACACAACAGCCCA
NOX1	Forward	CACAAGAAAAATCCTTGGGTCAA
	Reverse	GACAGCAGATTGCGACACACA
NOX2	Forward	CCTAAGATAGCGGTTGATGG
	Reverse	GACTTGAGAATGGATGCGAA
$p22^{phox}$	Forward	CGCTGGCGTCCGCCTGATCCTCA
	Reverse	ACGCACAGCCGCCAGTAGGTAGAT
$p40^{phox}$	Forward	TGAACAGCTTCCGGATGATG
	Reverse	TGAAGCCTCTCTTCTCCTCGAT
$p47^{phox}$	Forward	GTCAGATGAAAGCAAAGCGA
	Reverse	CATAGTTGGGCTCAGGGTCT
p67phox	Forward	ATCAGCCTCTGGAATGAAGGGG
	Reverse	GCAGCCAATGTTGAAGCAAATCC
NDUFS2	Forward	TGAGTTCTACGAGCGAGTGTCTGG
	Reverse	CGAAGCATCACTCCACTAAAACCAT
SDHD	Forward	TTCTTTCGTCGTCGTGGGTGG
	Reverse	GCTCGGTGACAAGTGTATGTGCTG
UQCR11	Forward	GTGATGTATCTGGGTGGTTGTGG
	Reverse	ACTGTGGCTTGCTCCTTCCTCT
COX4I1	Forward	GCTACCCTTTTCCGCTCCACG
	Reverse	AGCCAACATTCTGCCGCCACT
GAPDH	Forward	GCACCGTCAAGGCTGAGAAC
	Reverse	TGGTGAAGACGCCAGTGGA

Supplementary Table 2: Primers of rat used for quantitative real-time polymerase chain reaction (qRT-PCR)

Gene	Primers	Sequences (5′→3′)
CD31	Forward	TCTCTGCCTGATGAGTCTGC
	Reverse	CTCTGAGATGGCTGGTGTGG
vWF	Forward	GGTGGAGGAAGACCCCATTG
	Reverse	GATGTCCAGGTATGGCTCGG
a-SMA	Forward	TCATTGGAATGGAGTCGGCG
	Reverse	TTTGCGTTCTGGAGGAGCAATAA
Fap	Forward	TAGCAGTGGCTCCAGTCTCC
	Reverse	GCTCTTGCCATCACAGTTGA
IL-1β	Forward	GCCTCAAGGGGAAGAATCTATACC
	Reverse	GGGAACTGTGCAGACTCAAACT
IL-6	Forward	TCCTACCCCAACTTCCAATGCTC
	Reverse	TTGGATGGTCTTGGTCCTTAGCC
IL-10	Forward	ACTGGCTGGAGTGAAGACCA
	Reverse	CTTGGCAACCCAAGTAACCC
TNFβ1	Forward	ACGTCGTAGCAAACCACCAA
	Reverse	AAATGGCAAATCGGCTGACG
iNOS	Forward	CACAGAGGGCTCAAAGGAGG
	Reverse	AAAGTGGTAGCCACATCCCG
COX-2	Forward	CTTCGGGAGCACAACAGAGT
	Reverse	AAGTGGTAACCGCTCAGGTG
Actin	Forward	CCTCTATGCCAACACAGTGC
	Reverse	ATACTCCTGCTTGCTGATCC

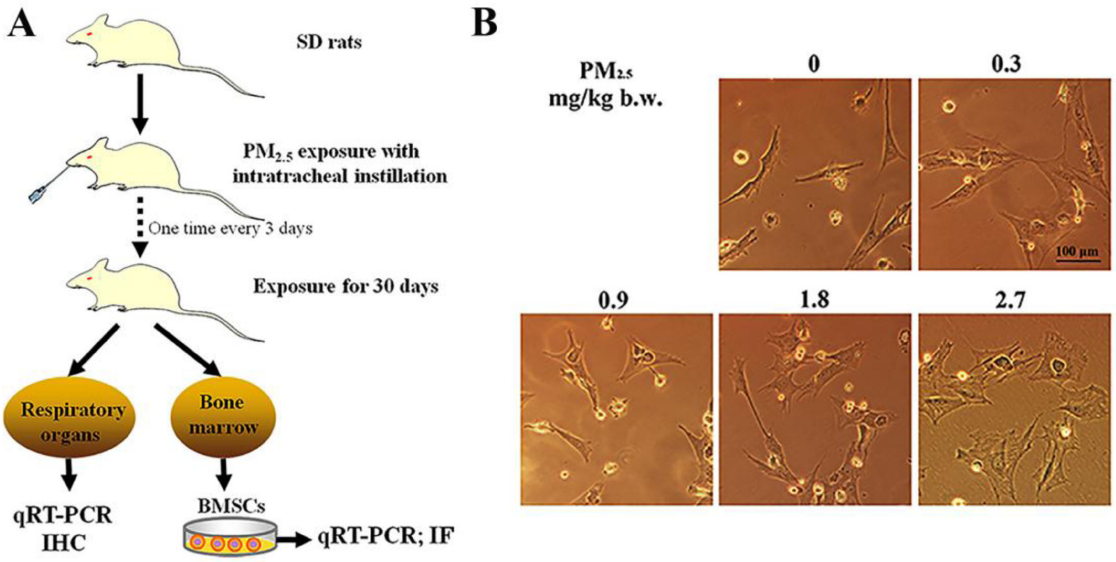

Supplementary Figure 1: The *in vivo* experimental protocol and effects of $PM_{2.5}$ on alterations of BMSCs morphology. Experimental protocol for $PM_{2.5}$-exposed SD rats. The morphology of BMSCs was observed using inverted microscope (20×). Scale bars are 100 μm.

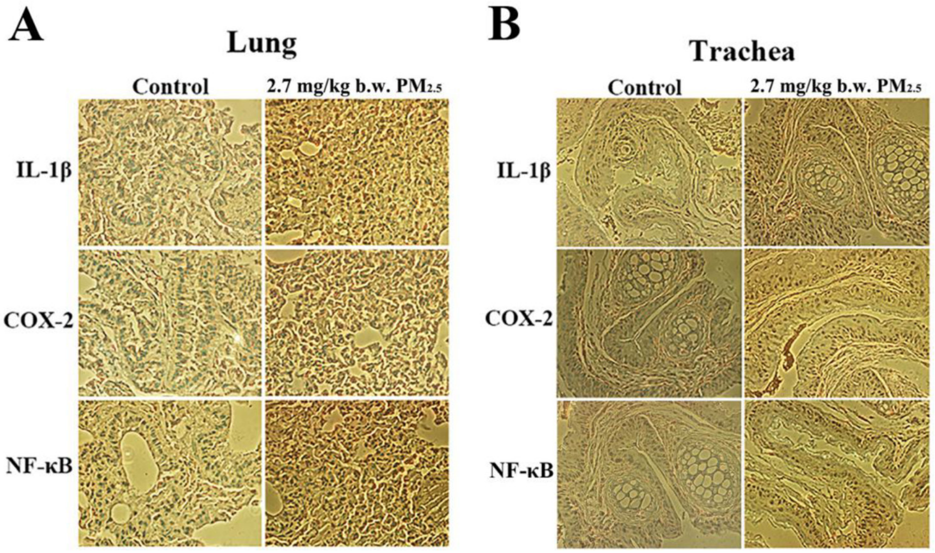

Supplementary Figure 2: Inflammatory response caused by $PM_{2.5}$ in lung and bronchial tissues. Immunohistochemistry analysis was carried out to determine the expressions of IL-1β, COX-2 and NF-κB in (**A**) lung tissues and (**B**) bronchial tissues. The image was observed using inverted microscope (20×).

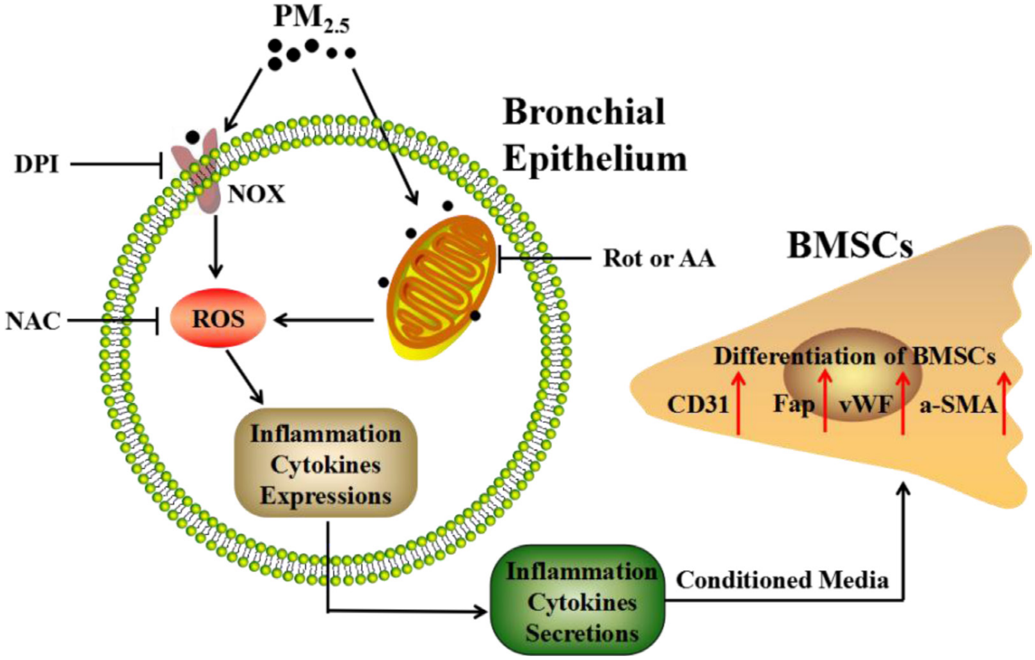

Supplementary Figure 3: The proposed model of $PM_{2.5}$-induced inflammatory cytokines leading to the differentiation of BMSCs into ELCs and CAFs. $PM_{2.5}$ exposure elevated the ROS generation via binding to cell membrane-bound NOX and causing mitochondrial disorder, which further provoked the inflammatory cytokine expressions and secretions in respiratory tract. Moreover, the secretions promoted the differentiation of BMSCs into ELCs and CAFs.

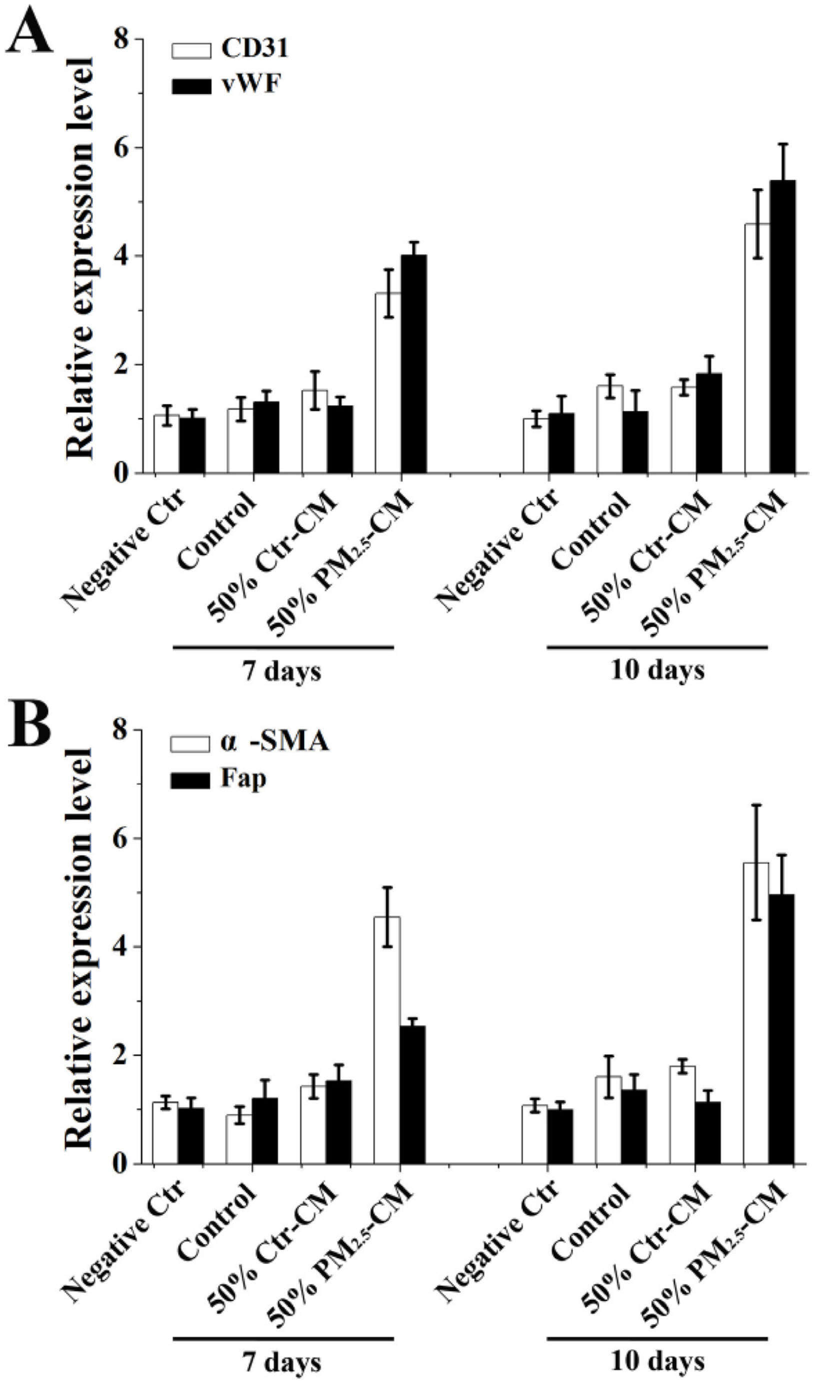

Supplementary Figure 4: The effects of CM from negative control, control and $PM_{2.5}$-exposed 16HBE cells on BMSCs differentiation. mRNA levels of (**A**) CD31, vWF, (**B**) a-SMA and Fap were measured in BMSCs. The values were showed as means ± SD of triplicate determinations. $^{*}p < 0.05$, $^{**}p < 0.01$, compared with control.
